# Antibacterial and anti-biofilm activity of radezolid against *Staphylococcus aureus* clinical isolates from China

**DOI:** 10.3389/fmicb.2023.1131178

**Published:** 2023-04-26

**Authors:** Cong Wang, Yanpeng Xiong, Chai Bao, Ying Wei, Zewen Wen, Xinyi Cao, Zhijian Yu, Xiangbing Deng, Guiqiu Li, Qiwen Deng

**Affiliations:** ^1^Department of Infectious Diseases and Shenzhen Key Lab of Endogenous Infection, Huazhong University of Science and Technology Union Shenzhen Hospital, Shenzhen, China; ^2^Department of Microbiology, The First Affiliated Hospital of Jiamusi University, Jiamusi, China; ^3^Department of Dermatology, Huazhong University of Science and Technology Union Shenzhen Hospital, Shenzhen, China; ^4^Heilongjiang Medical Service Management Evaluation Center, Harbin, Heilongjiang, China; ^5^Quality Control Center of Hospital Infection Management of Shenzhen, Huazhong University of Science and Technology Union Shenzhen Hospital, Shenzhen, China

**Keywords:** radezolid, *Staphylococcus aureus*, biofilm, quantitative proteomics, RT-PCR

## Abstract

Although the potent antibacterial ability of radezolid against *Staphylococcus aureus* has been widely reported worldwide, its antibacterial and anti-biofilm activity against the *S. aureus* clinical isolates from China remains elusive. In this study, the minimum inhibitory concentration (MIC) of radezolid was determined in *S. aureus* clinical isolates from China using the agar dilution method, and the relationship between radezolid susceptibility and ST distribution was also investigated. The anti-biofilm activity of radezolid against *S. aureus* was determined by a crystal violet assay and compared with that of linezolid and contezolid. The quantitative proteomics of *S. aureus* treated with radezolid was analyzed, and the genetic mutations in radezolid-induced resistant *S. aureus* were determined by whole-genome sequencing. The dynamic changes in transcriptional expression levels of several biofilm-related genes were analyzed by quantitative RT-PCR. Our data showed that radezolid MIC ranged from ≤0.125 to 0.5 mg/L, which was almost 1/4 × MIC of linezolid against *S. aureus*, indicating the greater antibacterial activity of radezolid than linezolid. The *S. aureus* clinical isolates with radezolid MICs of 0.5 mg/L were most widely distributed in ST239 of MRSA and ST7 of MSSA. Moreover, the more robust anti-biofilm activity of radezolid with subinhibitory concentrations (1/8 × MIC and 1/16 × MIC) was demonstrated against *S. aureus* when compared with that of contezolid and linezolid. Genetic mutations were found in *glmS*, 23S rRNA, and DUF1542 domain-containing protein in radezolid-induced resistant *S. aureus* selected by *in vitro* induction of drug exposure. Quantitative proteomic analysis of *S. aureus* indicated that the global expression of some biofilm-related and virulence-related proteins was downregulated. Quantitative RT-PCR further confirmed that the expressions of some downregulated biofilm-related proteins, including *sdrD, carA*, *sraP*, *hlgC*, *sasG*, *spa*, *sspP*, *fnbA*, and *oatA*, were decreased after 12 h and 24 h of exposure to radezolid. Conclusively, radezolid shows robust antibacterial and anti-biofilm activity against *S. aureus* clinical isolates from China when compared with contezolid and linezolid.

## Introduction

*Staphylococcus aureus* can live as commensal bacteria in the human body, and healthy people colonized with *S. aureus* are prone to developing an invasive infection. *S. aureus* is one of the major pathogens of hospital-acquired and community-acquired infections (Zipperer et al., 2016). *S. aureus* infection can cause a variety of infectious diseases, including skin and soft tissue infection (SSTI), endocarditis, and pneumonia. Recently, the development of antibiotic resistance in *S. aureus* has drawn attention worldwide. *S. aureus* clinical isolates with decreased susceptibility to current first-line antibiotics, such as linezolid, vancomycin, and daptomycin, have increasingly been reported in both methicillin-susceptible *S. aureus* (MSSA) and methicillin-resistant *S. aureus* (MRSA) (Li et al., 2020). The biofilm is a highly structured surface-associated microbial community that is enclosed in a self-produced protective extracellular matrix (Yan and Bassler, 2019). *S. aureus* biofilm forming on the surface of the medical device or tissue acts as a natural obstacle, which can hinder the penetration of antibiotics into the bacterial community and greatly reduce the effect of antimicrobial treatment. The gradual development of antibiotic resistance and biofilm formation have become two great challenges for improving the clinical outcome of the antimicrobial treatment of *S. aureus* infection. Thus, the discovery of novel antimicrobial agents is urgently needed for the antimicrobial treatment of multidrug-resistant *S. aureus* and biofilm-related infections.

Oxazolidinone antibiotics, including tedizolid, linezolid, and contezolid, have been approved for clinical application in China. Oxazolidinone class antibiotics were widely used in severe gram-positive bacterial infections by inhibiting the initial stage of bacterial protein synthesis, mainly through binding the 50S ribosome subunit (Quiles-Melero et al., 2013). In recent years, the gradual emergence of gram-positive bacteria that are resistant to linezolid or tedizolid, including *S. aureus, S. epidermidis, Enterococcus faecalis*, and *Enterococcus faecium*, has posed serious global challenges to the clinical application of oxazolidinone antibiotics (Dayan et al., 2016; Guo et al., 2019; Bakthavatchalam et al., 2021). Radezolid, a novel oxazolidinone antibacterial compound, has been approved by the FDA for clinical trials. Limited data demonstrated the excellent antibacterial effect of radezolid against gram-positive bacteria worldwide. At present, few studies of radezolid against *S. aureus* clinical isolates from China have been performed, and the anti-biofilm activity of radezolid remains unclear. Moreover, the difference in anti-biofilms against *S. aureus* clinical isolates from China among radezolid, linezolid, and contezolid needs to be further studied.

In this study, the antibacterial effect of radezolid on *S. aureus* clinical isolates from China was analyzed, and the anti-biofilm effect of radezolid with linezolid and contezolid was compared. The relationship between radezolid susceptibility and ST distribution in *S. aureus* clinical isolates from China was investigated. The effect of radezolid on the overall protein expression of *S. aureus* was mastered by quantitative proteomics, and whole-genome sequencing was used to determine the gene mutation of radezolid to induce drug-resistant *S. aureus.*

## Materials and methods

### Bacterial isolates

A total of 137 non-duplicated *S. aureus* clinical isolates were collected retrospectively in Huazhong University of Science and Technology Union Shenzhen Hospital (Grade A, Level III Hospital, 1,500 beds) from 01 January 2013 to 31 December 2014 from inpatients and outpatients (Supplementary Figure 1) and stored at −80°C. All clinical isolates were preliminarily identified by a Phoenix 100 automated microbiology system (BD, Franklin Lakes, NJ, USA). After stable passages with two generations, the species confirmation of *S. aureus* isolates was further determined by time-of-flight mass spectrometry (German IVD MALDI Biotyper). *S. aureus* ATCC 29213 was used as the quality control of antibiotic susceptibility strain.

### Antimicrobial susceptibility test

The oxazolidinone antibiotics (linezolid, radezolid, and contezolid) and other antibiotics used in this experiment were purchased from MedChemExpress (MCE, Shanghai, China). The minimum inhibitory concentration (MIC) of these antimicrobial agents was determined using the agar dilution method based on Clinical and Laboratory Standards Institute (CLSI) guidelines as described in our previous study (Zheng J. et al., 2020). While no standard breakpoint was recommended for radezolid against *S. aureus* in CLSI, in order to analyze the MIC distribution of radezolid, the MICs of radezolid against *S. aureus* were categorized into three levels, namely, ≤0.125, 0.25, and 0.5 mg/L, and those of linezolid were divided into ≤0.5, 1, 2, and 4 mg/L.

### Multilocus sequence typing (MLST)

The total genomic DNA of *S. aureus* isolates was extracted using a bacterial DNA extraction kit (TIANGEN, Beijing, China). Sequence typing (ST) of *S. aureus* clinical isolates was determined using the seven target housekeeping genes of MLST, including *arcC, aroE, glpF, gmk, pta, tpi*, and *yqi.* Primer synthesis and PCR amplification system were performed as described in previous reports (Bai et al., 2018; Zhang et al., 2018). The PCR products were sequenced, and the sequencing results were submitted to the pubmlst database for comparison to obtaining the ST type of the strain *S. aureus* in the MLST database.^1^

### *In vitro* induction of radezolid-resistant *S. aureus*

The parental *S. aureus* YUSA145 was used to select the radezolid-resistant isolates. YUSA145 single colony was inoculated into a TSB medium with an initial concentration of 0.25 mg/L of radezolid and linezolid. Then, radezolid or linezolid concentration for *in vitro* induction was increased one time after the bacteria with each concentration were cultured and passaged for five generations. After 35 days of induction, the single colony of radezolid-induced resistant isolate YUSA145RAD was chosen for the subsequent passage with three generations without antibiotics. Subsequently, the resolution of the YUSA145RAD strain was stored at −80°C for further use.

### Whole-genome sequencing

The chromosomal DNA of radezolid-resistant *S. aureus* isolate YUSA145RAD was extracted, and the whole-genome sequencing was performed on the Illumina HiSeq2500 sequencing platform of Novogene Co. Ltd. (Beijing, China). The reads were plotted against the reference genome of the YUSA145 strain in the bwa-mem software. Raw data for sequencing were uploaded to NCBI (accession number: PRJNA902154). SNP (single-nucleotide polymorphism), indel (insertion and deletion), and SV (structural variation) annotations between the parental YUSA145 and YUSA145RAD were compared with the genomic alignment results among samples using the MUMmer and LASTZ.

### Gene overexpression

The genetic mutation in the *glmS* gene was found in radezolid-resistant *S. aureus.* To verify the antimicrobial susceptibility and the genetic mutation, the coding sequence (CDS) of *glmS* was cloned into the BamH I and Ecor I sites of a PCN51 for His-tagged vector with the primers cgcGGATCCATGTGTGGAATTGTTGGTT and CCGgaattcTTATTCCACAGTAACTGATTTAG. Then, the pCN51-*glmS* was transformed into DC10B, and the extracted plasmid was transformed into SA113. The SA113 transformed with pCN51-*glmS*, containing the erythromycin resistance gene, was selected and identified. Moreover, the impact of overexpression vector pCN51-*glmS* on the radezolid susceptibility was further determined.

### Detection of *S. aureus* growth curve and biofilm formation

The influence of radezolid, contezolid, and linezolid with various concentrations (1/2 × MIC, 1/4 × MIC, 1/8 × MIC, and 1/16 × MIC) on the planktonic growth curve of the strain YUSA145 (ST239-MRSA clinical isolate) was investigated using the Bioscreen C system (Lab Systems Helsinki, Finland) at a wavelength of 600 nm as described in the previous report (Zhang et al., 2022).

The *Staphylococcus aureus* suspension under planktonic growth (OD≈1) was diluted with fresh TSBG in a ratio of 1:100 (Tryptone Soy Broth with 2% glucose) and inoculated in the microtiter plate. Subsequently, radezolid, contezolid, and linezolid (1/8 × MIC and 1/16 × MIC) were inoculated into the 96-well microtiter plate. After 24 h of incubation, the biofilm formation of *S. aureus* was determined using 96-well plate crystal violet staining at a wavelength of 570 nm, and the OD_570_ value represented the amount of *S. aureus* biofilm formation in each group. The eradication capacities of radezolid, contezolid, and linezolid were further evaluated after the mature biofilm of *S. aureus* formed. After 24 h of incubation, the *S. aureus* mature biofilm was formed in the 96-well microtiter plate. Then, the supernatant of the *S. aureus* mature biofilm was discarded, and the new culture media with radezolid, contezolid, and linezolid (8 × MIC) were added to the mature biofilm, respectively. After incubating for another 24 h, the remaining biofilm content was determined using the abovementioned crystal violet assay. The experiments were repeated at least three times.

### Confocal laser scanning microscope (CLSM)

The suspension of *S. aureus* strain YUSA145 (OD≈1) was inoculated into a confocal Petri dish containing 2 ml TSBG in a ratio of 1:200 with the concentrations of radezolid, contezolid, and linezolid (1/8 × MIC). After 24 h of incubation, the bacterial suspension was rinsed two times with PBS to remove the floating bacteria. Then, SYTO9/PI double staining was performed by light dyeing for 20 min, and the relative amounts of the live/dead bacterial cells were quantified and analyzed by CLSM (Grossman et al., 2021). All experiments were independently repeated three times.

### Quantitative analysis by nano LC-MS/MS

The biofilm formation of parental *S. aureus* YUSA145 isolates was cultured in an incubator at 37°C for 24 h, and the biofilm suspension was inoculated with the control and the radezolid (1/8 × MIC) for another 24 h. After the bacterial suspension was removed, the *S. aureus* biofilm cells were collected and homogenized with glass beads for three rounds and centrifuged at 4°C. The total protein of the bacterial supernatant was obtained, and the protein concentration was determined using the BCA protein assay kit (Beyotime Biotechnology, Shanghai, China) (Wen et al., 2022; Zhang et al., 2022). The 100 μg protein was pretreated for quantitative analysis by nano LC-MS/MS. After the sample was redissolved in mobile phase A (0.1% formic acid), 2 μl of the sample was loaded on a C18 pre-column (100 μm × 20 mm, Acclaim PepMap 100 C18). In mobile phase B, 80% acetonitrile and 0.1% formic acid were loaded. The column was coupled to a Q Exactive Plus mass spectrometer with a nanospray ionization (NSI) interface (ThermoFisher Scientific, Ohio, OH, USA). The MS1 full scan was performed in positive electrode mode, with an m/z range of 300–1,800 and a resolution of 70,000. The MS2 full scan was performed in collision-induced dissociation mode to further cleave the target ions and collect data. The Proteome Discoverer 2.4 was used to study the Uniprot proteome of *S. aureus*. The upregulation and downregulation of the proteins were determined by at least two technical replicates with a *p*-value of <0.05 and a two-fold cutoff value. The differential protein data were uploaded to OmicsBean for the Quick GO (Gene Ontology analysis), the KEGG Pathway (pathway analysis), and STRING (protein–protein interaction analysis).

### Quantitative real-time PCR and primer specificity

Briefly, YUSA145 (OD ≈ 1) was inoculated onto a 25 ml polypropylene culture plate containing 1/4 × MIC radezolid of fresh TSBG suspension and incubated at 37°C for 6, 12, and 24 h as in our previous studies (Zheng J. et al., 2020; Liu et al., 2022). Total RNA was extracted from planktonic bacteria and biofilms fluorescence quantitative PCR. The reference strain in the control group was not treated. The SYBR green PCR reagent (SYBR Premix ExTaq; TaKaRa Biotechnology, Dalian, China) was used to detect mRNA expression by RT-PCR in the Mastercycler Real Plex system (Eppendorf AG, Hamburg, Germany). The absorbance of RNA at OD_260_ and OD_280_ was determined using Nanodrop spectrophotometer ND-1000, and then the genomic DNA was digested according to the Takara reverse transcription kit. The 16S rRNA housekeeping gene served as a reference gene that standardized the transcription level. RT-PCR primer sequences are listed in Supplementary Table 1. The experiments were repeated three times.

### Statistical analysis

The data were analyzed using the student’s *t*-test. *P-*values of<0.05 were considered statistically significant. All data were analyzed using Statistical Product and Service Solutions (SPSS) version 16.0 (SPSS, Inc., Chicago, IL, United States).

## Results

### The *in vitro* antibacterial activity of radezolid against *S. aureus*

The antimicrobial susceptibility of radezolid against clinical isolates of MRSA and MSSA from China is shown in Table 1. The radezolid MIC against *S. aureus* ranged from ≤0.125 to 0.5 mg/L, and the frequencies of *S. aureus* with a radezolid MIC of ≤0.25 mg/L in MRSA and MSSA were 86.6% (71/82) and 92.7% (51/55), respectively. Moreover, the linezolid MIC ranged from ≤0.5 to 4 mg/L, and the MIC_50_/MIC_90_ of linezolid was also about four times that of radezolid, indicating the better *in vitro* antibacterial activity of radezolid when compared with linezolid. In the clinical isolates of MRSA and MSSA, the frequencies of chloramphenicol-resistant strains were 37.8% (31/82) and 25.5% (14/55). The frequency of chloramphenicol-resistant MRSA isolates with a radezolid MIC of 0.5 mg/L was 35.5% (11/31), while that of MSSA isolates was 28.6% (4/14). Previous reports have indicated that chloramphenicol could target the 50S ribosomes of the bacteria, which is similar to the target of linezolid and clindamycin in bacteria, and the correlation of chloramphenicol susceptibility with that of radezolid needs to be further studied.

**TABLE 1:** Relationship of the MIC values of radezolid and linezolid with the antimicrobial susceptibility of some commonly used antibiotics against MSSA and MRSA.

Organism antibiotic		RZD MIC distribution (mg/L)				LZD MIC distribution (mg/L)				
		**≤0.125**	**0.25**	**0.5**	**MIC_50_/MIC_90_**	**≤0.5**	**1**	**2**	**4**	**MIC_50_/MIC_90_**
**MRSA**										
AK	S/I	8	15	2	0.25/0.25	7	9	2	6	1/4
	R	28	20	9	0.25/0.5	14	31	4	9	1/2
TC	S/I	5	9	3	0.25/0.5	6	3	2	8	1/4
	R	31	26	8	0.25/0.5	15	37	4	7	1/2
CL	S/I	18	15	5	0.25/0.5	7	21	4	7	1/4
	R	18	20	6	0.25/0.5	14	19	2	8	1/2
CHL	S/I	25	26	0	0.25/0.25	20	27	3	5	1/1
	R	11	9	11	0.25/0.5	1	13	3	10	2/4
AK	S/I	10	30	1	0.25/0.25	24	12	3	1	≤ 0.5/2
	R	5	6	3	0.25/0.5	6	2	5	2	1/2
TC	S/I	10	29	2	0.25/0.25	29	8	4	0	≤ 0.5/1
	R	5	7	2	0.25/0.5	1	6	4	3	1/2
CL	S/I	11	27	3	0.25/0.25	27	9	5	2	≤ 0.5/2
	R	4	9	1	0.25/0.25	3	5	3	1	1/2
CHL	S/I	13	28	0	0.25/0.25	29	10	2	0	≤0.5/1
	R	2	8	4	0.25/0.5	1	4	6	3	2/4

MRSA, *n* = 82; MSSA, *n* = 55; S, susceptible; I, intermediate; R, resistant; AK, amikacin; Tc, tetracycline; CL, ciprofloxacin; CHL, chloramphenicol; LZD, linezolid; RZD, radezolid; MIC, minimum inhibitory concentration; MIC_50_, 50% minimum inhibitory concentration; MIC_90_, 90% minimum inhibitory concentration.

### Relationship between ST distribution and radezolid MIC in *S. aureus* clinical isolates

Sequence type and radezolid MIC of *S. aureus* clinical isolates from China are shown in Table 2. The frequencies of ST239 and ST59 in MRSA were 70.7% (58/82) and 18.3% (15/82), respectively. The frequencies of ST7 and ST398 in MSSA were 45.4% (25/55) and 30.9% (17/55), respectively. The frequencies of a radezolid MIC of 0.5 mg/L were observed in 10.3% (6/58) of ST239-MRSA and 33.3% (5/15) of ST59-MRSA. Moreover, the frequency of ST7-MSSA clinical isolates with a radezolid MIC of 0.5 mg/L in the total number of ST7-MSSA was 12% (3/25) and the frequency of ST398-MSSA in the total number of ST398-MSSA was 5.9% (1/17). Notably, the frequency of ST239 and ST59 in MRSA clinical isolates with a radezolid MIC of 0.5 mg/L was 91% (11/12). Conversely, the frequency of ST7 and ST398 in MSSA isolates with a radezolid MIC of 0.5 mg/L was 80% (4/5). However, the proportion of other MICs of radezolid in MRSA and MSSA had the same trend as high MICs. Our data suggested that there is no association between a high radezolid MIC and ST.

**TABLE 2:** Relationship between STs and the MIC value of radezolid or linezolid in *S. aureus.*

Organism	MLST	*N*	RZD MIC distribution (mg/L)			LZD MIC distribution (mg/L)		
			**≤0.125**	**0.25**	**0.5**	**≤0.5**	**1**	**≥2**
MRSA	ST239	58	24	28	6	20	28	10
	ST59	15	5	5	5	0	6	9
	ST1	6	5	1	0	1	4	1
	others	3	1	1	1	0	2	1
MSSA	ST7	25	8	14	3	12	6	7
	ST398	17	5	11	1	9	5	3
	ST59	4	1	3	0	2	2	0
	Others	9	1	7	1	7	1	1

LZD, linezolid; RZD, radezolid; MIC, minimum inhibitory concentration; ST, sequence type.

### Genetic mutation of radezolid-induced resistant *S. aureus* by whole-genome sequencing

**FIGURE 1:**
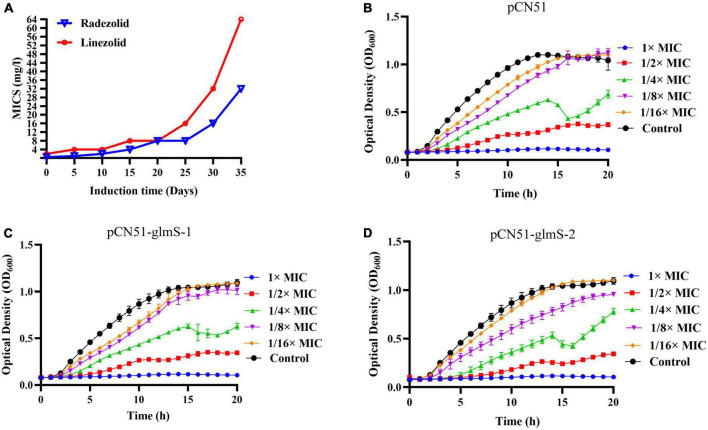
**(A)** *Staphylococcus aureus* YUSA145 resistance to radezolid *in vitro* induction. **(B–D)** The planktonic growth curve of *S. aureus* SA113 with *glmS* overexpression and the control transfected with a pCN51 empty vector. The presented data were the average of three independent experiments (mean ± SD).

After 35 days of continuous passages of YUSA145 *in vitro* under the pressure of radezolid, the resistant *S. aureus* strain YUSA145RAD induced by radezolid was selected and identified. The dynamic changes between the MICs of radezolid and linezolid in radezolid-induced resistant *S. aureus* strain YUSA145RAD are shown in Figure 1A, suggesting the MICs of radezolid and linezolid in radezolid-resistant *S. aureus* strain YUSA145RAD were 32 mg/L and 64 mg/L, respectively. In addition, the MICs of radezolid and linezolid in linezolid-induced resistant *S. aureus* strain MS4LZD were 16 and 32 mg/L, respectively (Supplementary Table 2). The whole-genome sequencing of radezolid-resistant *S. aureus* strain YUSA145RAD and linezolid-induced resistant *S. aureus* MS4LZD was performed, and our data indicated the non-synonymous mutations were determined in three functional genes of radezolid-resistant *S. aureus* strain YUSA145RAD. The genetic nonsense mutations of YUSA145RAD in DUF1542 domain-containing protein, *glutamine-fructose-6-phosphatet ransaminase* (*glmS*) and *23S ribosomal RNA* are listed in Table 3. The four genetic mutation points in *23S ribosomal RNA* were found in MS4LZD. Then, *S. aureus* was transfected with the overexpression vector of glmS to evaluate the impact of *glmS* on the radezolid susceptibility, suggesting that the overexpression of the gene *glmS* did not change the radezolid MIC of SA113 (Supplementary Table 3). In addition, the planktonic growth of *glmS* overexpression *S. aureus* strains (pcn51-*glmS*-1 and pcn51-*glmS-*2) under the subinhibitory concentration of radezolid showed no difference when compared with the growth of the pCN51 empty vector (Figures 1B–D), indicating the *glmS* mutation might not impact the radezolid susceptibility in *S. aureus*.

**TABLE 3:** Genetic mutations in YUSA145RAD and MS4LZD were detected by whole-genome sequencing.

Strain	NA mutations	AA mutations	Ref_gene_product
YUSA145RAD	C5132T	A1711V	DUF1542 domain-containing protein
	C242T	A81V	glutamine-fructose-6phosphate transaminase (*glmS*)
	T158A	L53H	23S ribosomal RNA
MS4LZD	G379T	A127S	23S ribosomal RNA
	A1991G	N664S	23S ribosomal RNA
	G2043C	E681D	23S ribosomal RNA
	C2051T	S684F	23S ribosomal RNA

### Significant inhibition of *S. aureus* biofilm formation by radezolid

**FIGURE 2:**
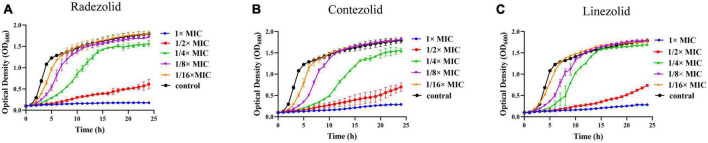
Effect of oxazolidinone antibiotics on the planktonic growth of *S. aureus*. The optical density (OD_600_) of *S. aureus* YUSA145 at subinhibitory concentrations (1/2 × MIC, 1/4 × MIC, 1/8 × MIC, and 1/16 × MIC) of radezolid **(A)**, contezolid **(B)**, and linezolid **(C)** was determined using the automatic growth curve method. The presented data were the average of three independent experiments (mean ± SD).

**FIGURE 3:**
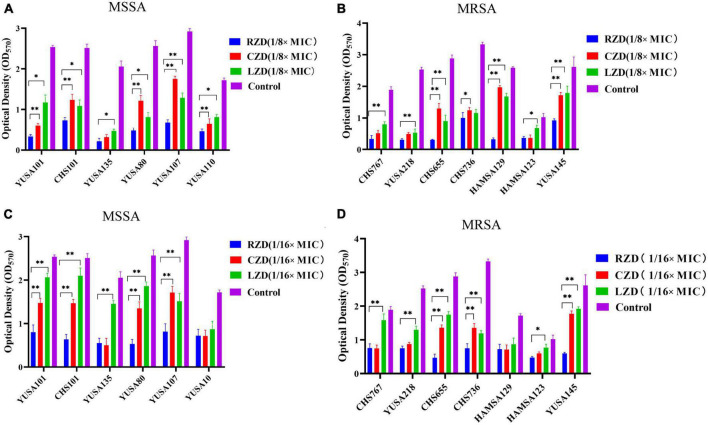
The inhibitory effect of subinhibitory concentrations (1/8 × MIC and 1/16 × MIC) of radezolid, contezolid, and linezolid on biofilm formation. The anti-biofilm effect of radezolid, contezolid, and linezolid against MSSA **(A)** and MRSA **(B)** isolates was assessed at 1/8 × MIC. The anti-biofilm effect of radezolid, contezolid, and linezolid against MSSA **(C)** and MRSA **(D)** isolates was assessed at 1/16 × MIC. The presented data were the average of three independent experiments (mean ± SD). ^**^P < 0.001, *P < 0.05 (Student’s *t*-test). MIC, minimum inhibitory concentration; RZD, radezolid; CZD, contezolid; LZD, linezolid.

**FIGURE 4:**
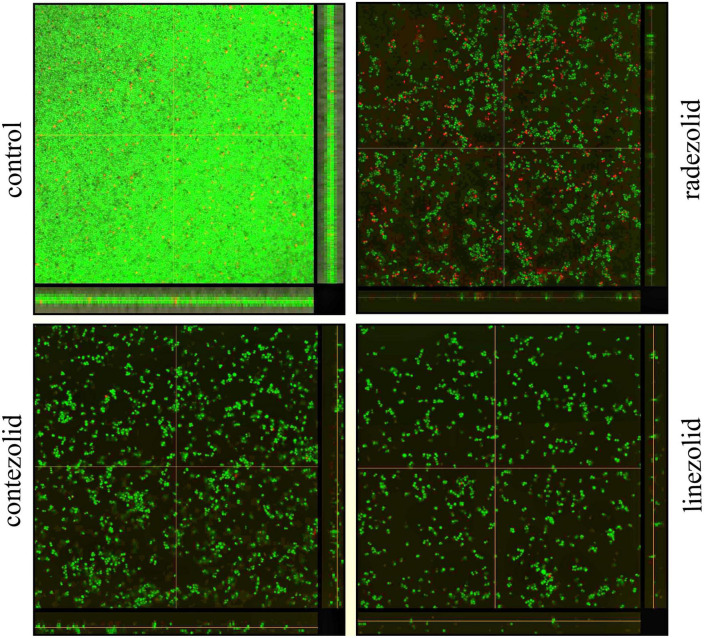
The inhibitory effects of radezolid, contezolid, and linezolid on the biofilm inhibition of *S. aureus* by laser scanning confocal microscopy.

The inhibition of *S. aureus* biofilm formation was investigated with subinhibitory concentrations of radezolid, contezolid, and linezolid (1/2 × MIC, 1/4 × MIC, 1/8 × MIC, and 1/16 × MIC) (Figures 2A–C). The automatic planktonic growth curve indicated that concentrations of 1/8 × MIC and 1/16 × MIC had no significant inhibition of *S. aureus* planktonic cells. Therefore, the effects of oxazolidinones at concentrations of 1/8 × MIC and 1/16 × MIC on *S. aureus* biofilm were investigated. Radezolid with a concentration of 1/8 × MIC showed better anti-biofilm activity in six MSSA isolates than linezolid, and it had a more robust inhibitory effect against biofilm formation in five MSSA isolates when compared with contezolid (shown in Figure 3A). Radezolid with a concentration of 1/16 × MIC inhibited more significantly the biofilm formation in five MSSA isolates than linezolid, and the anti-biofilm activity of radezolid was significantly better than that of contezolid in four MSSA isolates (shown in Figure 3C). This trend was also observed for MRSA (Figures 3B, D). Radezolid with a concentration of 1/8× or 1/16× of the MIC efficiently inhibited the biofilm formation in seven MRSA isolates when compared with contezolid and linezolid. The robust inhibitory activity of *S. aureus* biofilm formation by subinhibitory concentrations of radezolid was further confirmed by laser confocal scanning microscopy (Figure 4), suggesting a more significant decrease in the number of bacterial cells by radezolid when compared with contezolid and linezolid. However, radezolid, contezolid, and linezolid had no scavenging effect on the mature biofilm of seven *S. aureus* isolates (Supplementary Figure 2).

### Global proteomic analysis of *S. aureus* treated with radezolid

**FIGURE 5:**
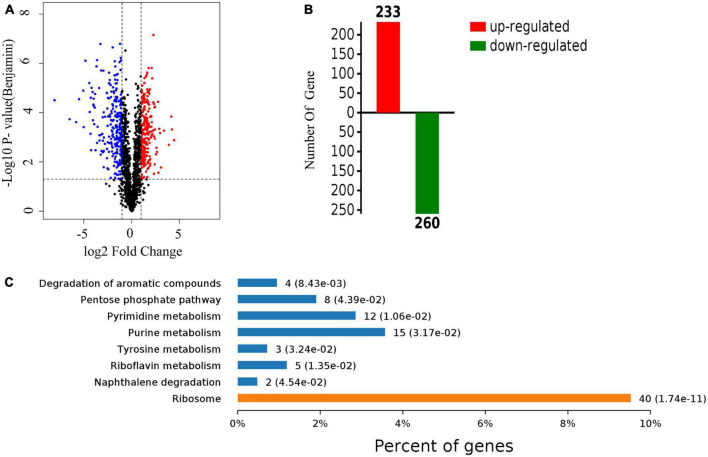
The proteomic analysis of *S. aureus* treated with radezolid. **(A)** Volcano plots show the comparison of the proteomic analysis of *S. aureus* treated with radezolid (1/8 × MIC). Blue dots represent the decreased levels of *S. aureus* proteins caused by radezolid exposure. Red dots represent the upregulated levels of *S. aureus* proteins caused by radezolid exposure. The *P*-value was calculated using a two-sided, two-sample *t*-test; three independent experiments were performed per group. The data of the protein expression levels were calculated from the average value. **(B)** A number of differentially expressed proteins in *S. aureus* with radezolid and control, respectively. **(C)** KEGG pathway in *S. aureus* with radezolid and control, respectively.

**FIGURE 6:**
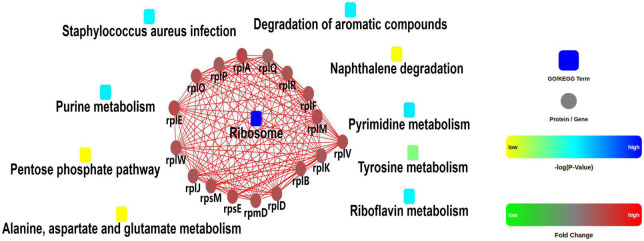
Protein–protein interaction network analysis for the differentially expressed proteins between the control groups and radezolid-treated groups.

A quantitative proteomic analysis of *S. aureus* treated with radezolid or as a control was performed, and a total of 1,448 proteins was tested for the quantification analysis. The quantitative level of the quantified proteins with ≥2-fold changes was defined as significantly different (*P* < 0.05). Among the total 1,448 proteins, 493 were classified as significantly differentially expressed ones, of which 233 proteins (Supplementary Table 4) were upregulated and 260 proteins (Supplementary Table 5) were downregulated (Figures 5A, B). The quantitative proteins were identified and uploaded to the OmicsBean website. The Kyoto Encyclopedia of Genes and Genomes (KEGG) pathway (Figure 5C) and protein–protein interaction (PPI) network (Figure 6) were obtained. Consistent with the global analysis of the KEGG pathway, the category of significantly expressed proteins in the PPI network was enriched for those correlated with the ribosome. Notably, the most significant type and function of proteins responding to radezolid stress were ribosomal proteins. The upregulated expression of *mutS2, ruvB, dnaE, nfo, ruvA, xseA, SAOUHSC_01744, ung*, and other genes involved in DNA repair indicated that these results might be related to the compensatory activities of bacteria in response to the environment. The protein levels of some biofilm-related factors and adhesion proteins, such as *hlgC, sspP, sdrD, fnbA*, and *sdrC*, were found to be downregulated in the radezolid-treated *S. aureus*.

### The transcriptional levels of some biofilm-related genes in radezolid-treated *S. aureus*

To verify the impact of radezolid on the transcriptional levels of biofilm-related genes in *S. aureus* treated with radezolid, the mRNA expression levels of biofilm-related genes were determined at 6, 12, and 24 h after exposure to 1/8 × MIC of radezolid. Previous studies have indicated that some biofilm-related factors, including gamma-hemolysin component (hlgc), o-acetyltransferase (oatA), staphopain (sspP), serine-aspartate repeat-containing proteins (sdrD and sdrC), immunoglobulin-binding protein (sbi), and bifunctional autolysin (atl), participated in the virulence of *S. aureus*, and the proteomic analysis showed their downregulation (Supplementary Table 4). The transcriptional RNA levels of *sdrD*, *carA*, *sraP*, *hlgC*, *sagG, spa, icaB, oatA, sspP*, and *fnbA* were markedly decreased when the *S. aureus* isolates were treated with radezolid for 12 h. The transcriptional levels of these biofilm-related genes in *S. aureus* isolates, including *sdrD, carA, sraP, hlgC, sagG, spa, icaB, oatA, sspP*, and *fnbA*, were generally significantly decreased for 24 h (Table 4).

**TABLE 4:** The RNA expression levels of biofilm-related genes in *S. aureus* YUSA145 treated with radezolid.

Biofilm formation-related genes	YUSA145		
	**6 h**	**12 h**	**24 h**
*sdrD*	4.59	1.78	0.033
*carA*	33.413	1.58	0.069
*sraP*	6.69	0.57	0.00196
*hlgC*	8.75	0.306	0.0617
*icaB*	8.02	0.31	0.00152
*sagG*	1.62	0.34	0.018
*Spa*	4.84	0.817	0.55
*sspP*	3.41	1.64	0.818
*oatA*	14.67	2.42	0.848
*fnbA*	20.45	1.67	0.034

Radezolid was used at 1/8 × MIC. The RNA levels were detected by RT-PCR, with an untreated isolate as the reference strain (mRNA level = 1.0).

## Discussion

Refractory infection caused by *S. aureus* is mainly due to antibiotic resistance and biofilm formation. Oxazolidinones, including linezolid, contezolid, and tedizolid, are applied as the first-line drug for the antimicrobial treatment of gram-positive bacterial infections. Multiple reports have shown the excellent antibacterial activity of radezolid against the planktonic cells of gram-positive bacteria (Moellering, 2014; Zheng J. et al., 2020; Wang et al., 2021). A previous study shows that radezolid still has potent antibacterial activity against *E. faecalis* linezolid non-susceptible strains, with the MIC of radezolid being 2–8 times lower than that of linezolid (Zheng J. et al., 2020). Our data further indicated that radezolid had stronger antibacterial activity than linezolid and remained the drug with excellent bactericidal activity against *S. aureus* clinical isolates from China. In fact, we determined the MIC of contezolid against a minority of *S. aureus* isolates, and the antibacterial activity of contezolid was not stronger than that of linezolid among these selected strains (Supplementary Table 6). In a previous study, it was found that clinical isolates of *E. faecium* with radezolid MIC ≥ 0.5 mgl/L were clustered in ST78 and ST18. However, our study did not find that the MIC of radezolid was related to ST in these clinical isolates of *S. aureus.*

Linezolid can exert an antibacterial effect mainly by inhibiting protein synthesis and binding to the peptidyl transferase center (PTC) of the bacterial 50S ribosomal subunit. One of the main mechanisms of linezolid resistance is mediated by the genetic mutation in the V region of the bacterial 23S rRNA, which leads to the decreased affinity of the linezolid for the target site. A previous study has confirmed that radezolid resistance can be explained by genetic mutations in V regions of 23S rRNA and 50S ribosome-related proteins (Xu et al., 2020). Our previous studies have indicated the genetic mutation in 23S rRNA of *E. faecalis, E. faecium*, and *S. agalactiae* was induced by radezolid pressure with *in vitro* induction (Xu et al., 2020; Zheng J. et al., 2020; Zheng J. X. et al., 2020). Here, the cross-resistance between linezolid and radezolid was found in the radezolid-resistant *S. aureus* strains. The genetic mutations of the V domain of 23S rRNA were found in both radezolid- and linezolid-induced resistant *S. aureus* strains, further demonstrating the close correlation of radezolid resistance with genetic mutation of the 23S rRNA V domain. Moreover, our data suggested that continuous radezolid exposure could result in its cross-resistance to linezolid.

Besides the mutational points of 50S ribosome subunits, several reports also indicate the additional complicated mechanisms of linezolid resistance in *S. pneumonia* (Feng et al., 2009; Billal et al., 2011). The genetic mutation of the potential target sites of antibiotics in the *in vitro* induction bacterial isolates with antibiotic resistance can often be determined by whole-genome sequencing. In this study, in addition to the genetic mutation of 23S rRNA, the genetic mutations in *glmS* and DUF1542 domain protein in the radezolid-induced resistant *S. aureus* were also found. The correlation of genetic mutations in *glmS* and DUF1542 domain proteins with linezolid or radezolid resistance has not been reported. Seldom studies indicated the critical role of DUF1542 domain protein in the biofilm formation and growth of *S. aureus*, hypothesizing no impact of this protein on the radezolid susceptibility. Glucose-6-phosphate synthase (*glmS)* is a key enzyme for catalyzing the metabolism of hexosamine, which is the final product of UDP-N-acetylglucosamine-6P in the metabolic pathway of *S. aureus* and is considered an important component of bacterial cell walls (Milewski, 2002). Therefore, the overexpression of *glmS* on the radezolid susceptibility in *S. aureus* was determined, and our data indicated that bacterial growth and the MIC of radezolid could not be impacted by the overexpression of level *glmS*. The relationship between *glmS* and anti-biofilm activity and radezolid susceptibility needs to be further studied.

Biofilm formation is often explained as a three-dimensional bacterial aggregation and can resist environmental and antibiotic pressure. Biofilm-embedded bacterial cells often show higher antibiotic resistance than that in the planktonic condition. Previous studies have demonstrated a stronger ability to inhibit *E. faecalis* biofilms than linezolid (Zheng J. et al., 2020). Our data indicated the significant inhibition of the biofilm by 1/8 × MIC and 1/16 × MIC of radezolid, contezolid, and linezolid, and worthy of our attention is the inhibitory effect of radezolid against the *S. aureus* biofilm formation was stronger than that of contezolid and linezolid. Quantitative proteomic analysis indicated that the inhibition of radezolid on *S. aureus* biofilms can be partly explained by significantly reducing the protein levels of biofilm-related genes, including *icaB, spa, fnbA*, and *sasG*, after radezolid exposure. The accumulation of biofilm formation of *S. aureus* mainly depends on the synthesis and function of polysaccharide intercellular adhesion (PIA) molecules encoded by the *icaADBC* gene (Hait et al., 2021). The *icaR* gene negatively regulates the expression of *icaABCD*. *IcaB* is a secreted protein that plays an important role in *S. aures* adhesion to host cells (Arciola et al., 2015). In addition, numerous reports support ica-independent biofilms can also be found, suggesting the complicated mechanism of biofilm formation in *S. aureus.* The functional protein encoded by the *sasG* gene promotes biofilm formation in *S. aureus* through the pathway independent of PIA (Corrigan et al., 2007). Protein A (spa) is an important component of *S. aureus* biofilm and promotes the induction and development of biofilm (Merino et al., 2009). *FnbA* can promote the intercellular accumulation and biofilm formation of *S. aureus* through binding extracellular matrix proteins (O’Neill et al., 2008). A recent study has shown that linezolid reduces *S. aureus* biofilm formation by affecting *IcaA* and *IcaB* proteins (Bi et al., 2022). Therefore, the global proteomic response of *S. aureus* by radezolid supported the inhibition of radezolid on *S. aureus* biofilms, which is partly explained by significantly impacting some important protein expression of biofilm-related genes.

Virulence-related factors in *S. aureus* contribute to bacterial colonization, host tissue invasion, and biofilm adhesion (Jenkins et al., 2015). Several previous studies have shown linezolid at sub-MIC concentrations can reduce the expression of some important virulence-related factors, including alpha-haemolysin (*hla)*, delta-haemolysin (*hld*), enterotoxin A (*sea*), bifunctional autolysin, and autolysin in *S. aureus* (Gemmell and Ford, 2002; Bernardo et al., 2004). Here, quantitative RT-PCR indicated that the transcription levels of some biofilm-related genes were significantly decreased in *S. aureus* after radezolid exposure at 12 h and 24 h in our study, including *icaB, sdrD, carA, sraP, hlgC, sasG, spa, sspP, fnbA*, and *oatA*. In fact, these biofilm-related genes also participate in the virulence of *S. aureus.* Therefore, the anti-virulence activity of radezolid should be further studied in the future.

## Conclusion

In summary, radezolid has a strong inhibitory effect on the planktonic growth and biofilm formation of *S. aureus* clinical isolates from China. Our data suggested the radezolid MIC ranged from ≤0.125 to 0.5 mg/L and was almost 1/4 that of linezolid, indicating the greater antibacterial activity of radezolid than linezolid. The clinical isolates of *S. aureus* with a radezolid MIC of 0.5 mg/L were mainly distributed in ST239 of MRSA and ST7 of MSSA. Radezolid with a sub-MIC can significantly inhibit the biofilm formation of *S. aureus* compared to linezolid. Moreover, the rapid emergence of radezolid resistance was found by *in vitro* induction. The inhibition of radezolid against biofilm formation might be explained by impacting the protein expression levels of some biofilm-related genes and virulence-related genes.

## Data availability statement

The original contributions presented in this study are included in the article/Supplementary material, further inquiries can be directed to the corresponding authors.

## Ethics statement

All procedures involving human participants were performed in accordance with the Ethical Standards of Shenzhen University School of Medicine and with the 1964 Helsinki Declaration and its later amendments, and this study was approved by the Ethics Committee of the 6th Affiliated Hospital of Shenzhen University Health Science Center.

## Author contributions

CW participated in the design of the study, analyzed the proteomics data, and drafted the manuscript. YX carried out a biofilm assay by crystal violet staining and cytotoxicity assay. CB participated in the biofilm assay. YW participated in the whole-genome sequencing data analysis. ZW participated in the proteomics data analysis and gene overexpression. XC performed the MIC assay. ZY and XD reviewed the manuscript. QD and GL designed the study, participated in the data analysis, and provided the critical revisions of the manuscript for valuable intellectual content. All authors contributed to the article and approved the submitted version.

## Footnotes

1
https://pubmlst.org/
