# Supplementary material for: Antibacterial and anti-biofilm activity of radezolid against Staphylococcus aureus clinical isolates from China
Source: Front Microbiol. 2023 Apr 26;14:1131178. doi: 10.3389/fmicb.2023.1131178 (PMC10169660; doi:10.3389/fmicb.2023.1131178)
Supplement: Supplementary file 1 [file Data_Sheet_1.PDF]

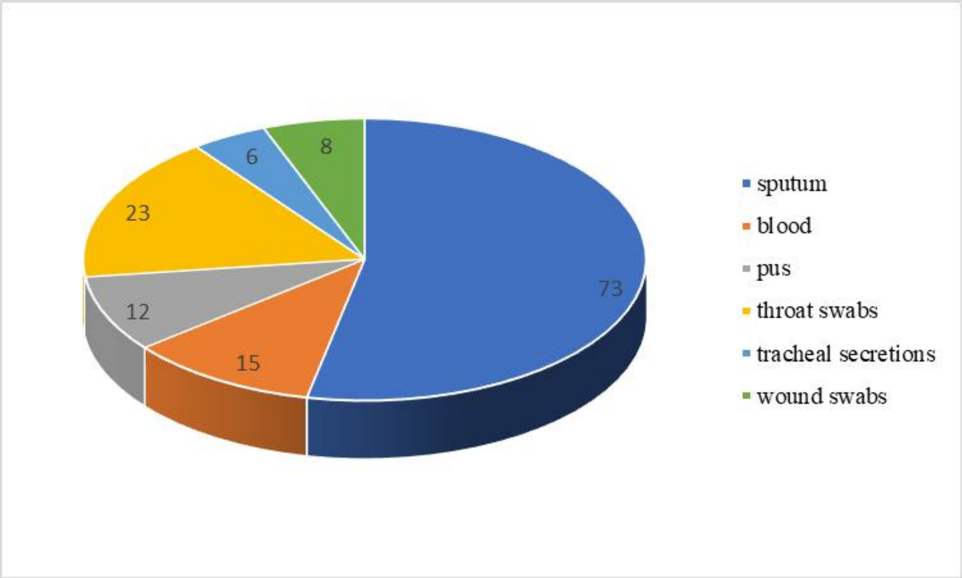

Supplement Figure 1 The source of 137 non-repetitive *S. aureus*.

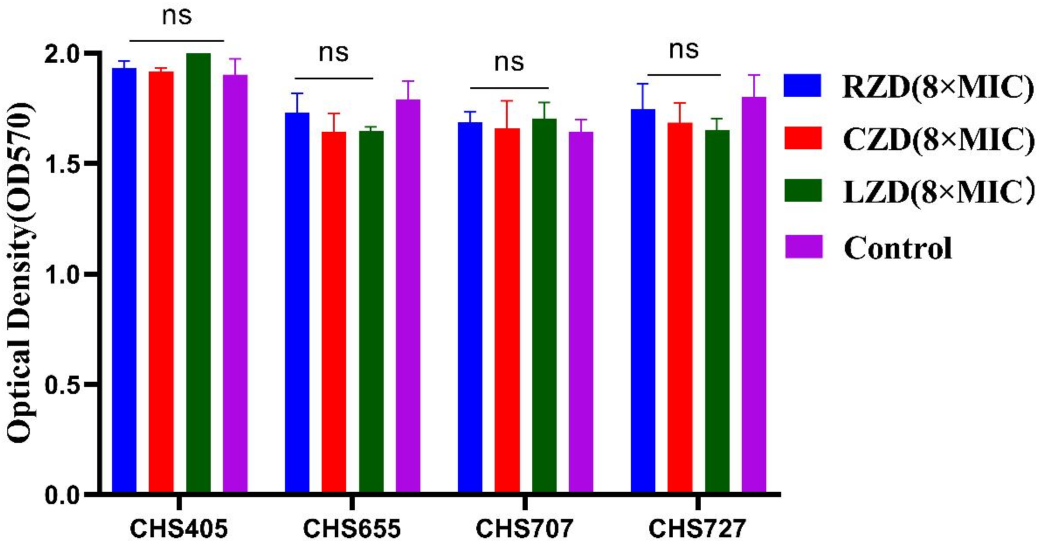

Supplement Figure 2. Impact of radezolid, contezolid, linezolid (all 8x MIC) on the eradication of mature biofilm of *S. aureus***.**

Supplementary table 1. The primers sequence of quantitative RT-PCR.

gene	Sequence (5'-3')	Product size
sdrD-F	GCAGATGGTGGCGAAGTTGACG	157bp
sdrD-R	CACTGTCTGAGTCTGAGTCGCTGT	
carA-F	CGAGTGCGCATCCAAGTAAT	130bp
carA-R	AACACACCGTGTTGTCGAAT	
sraP-F	AAGCACAACGCCATCTTCGGT	123bp
sraP-R	GTCGTCGCTGCTGACGCAAA	
16srna-F	CCTACGGGAGGCAGCAG	199bp
16srna-R	ATTACCGCGGCTGCTGG	
sasG-F	AGACACTGCACCACAGGGTGT	178bp
SasG-R	TGTCTCTGCGTTATGAGTGACTGACG	
spa-F	AGCGCTTTGGCTTGGGTCAT	144bp
spa-R	GAATCTCAAGCACCGAAAGCGGAT	
hlgC-F	ATTTCCAATCAGCCCCATCACTCG	258bp
hlgC-R	CAAGAGGTGGTAACTCACTGTCTGG	
icaB-F	CACAGGTCATGTTGGGGAAGAAAAC	350bp
icaB-R	AATCATTGGAGTTCGGAGTGACTGC	
sspP-F	TTGGACAAACTCAGGGAAGAAGTCCA	171bp
sspP-R	ATTTAGCGTTCCCAACAACTGCCA	
oatA-F	AGCAAGAGGATAAGCAGACAGCGA	160bp
oatA-R	GCATCAACGAGTTGCCGTCCA	
fnbA-F	GGCACAGCCAAGAACGGCAT	

fnbA-R	ACCCGTTTCCACTTTCGCGTT	100bp

Supplementary table 2. The MIC values of radezolid and linezolid in radezolid or linezolid-induced *S. aureus*.

Strains	Radezolid MIC (mg/L)	Linezolid MIC (mg/L)
YUSA145RAD	32	64
MS4L100	16	32

Supplementary table 3. The radezolid MIC of *S. aureus* SA113 transfected with *glms* and pCN51 empty-vector control

**Strains**	**MIC of value radezolid(mg/L)**
Pcn51	0.25
Pcn51-1	0.25
Pcn51-2	0.25

Supplementary table 4. The differentially expressed proteins with the up-regulated level by quantitative proteomic analysis in *S. aureus* treated with radezolid.

ID	Log2FoldChange	Description
Q2FZW2	2.39	NifU domain-containing protein
Q2FXV2	1.41	Staphylococcal protein
Q2FW38	1.71	50S ribosomal protein L13
P48860	1.09	50S ribosomal protein L7/L12
Q2FW18	1.86	50S ribosomal protein L5
P0A0G2	1.61	50S ribosomal protein L30
Q2G066	1.11	Bacillithiol system redox-active protein YtxJ
Q2FZ25	1.36	30S ribosomal protein S2
Q2FW20	1.74	30S ribosomal protein S8
Q2FWD8	1.28	50S ribosomal protein L31 type B
Q2FW16	2.07	50S ribosomal protein L14
Q2FUQ9	3.38	Cold shock protein CspA
Q2FW23	1.84	30S ribosomal protein S5
Q2FW10	1.95	30S ribosomal protein S19
Q2FW21	1.51	50S ribosomal protein L6
Q2G0N1	1.28	Elongation factor G
Q2G0V0	1.67	Lipoprotein
Q2FW30	1.68	30S ribosomal protein S13
Q2FW08	1.46	50S ribosomal protein L23
Q2FZ45	1.34	30S ribosomal protein S16
Q2G0N7	1.62	MTS domain-containing protein
Q2FW11	1.69	50S ribosomal protein L22
Q2G298	1.91	Ribosomal silencing factor RsfS
Q2FW12	1.48	30S ribosomal protein S3
Q2FXW6	1.71	Uridine kinase
Q2G1K7	1.57	non-specific protein-tyrosine kinase
Q2G0B1	1.59	HTH-type transcriptional regulator MgrA
Q2FW14	1.66	50S ribosomal protein L29
P0A0F8	1.64	50S ribosomal protein L15
Q2G1K2	1.16	UDP-N-acetylglucosamine 2-epimerase
Q2G2L6	2.77	DUF2188 domain-containing protein
Q2G2T0	1.04	YtxH domain-containing protein
Q2FZ28	1.32	ATP-dependent protease ATPase subunit HslU
Q2FW22	1.5	50S ribosomal protein L18
Q2FXK6	2.34	30S ribosomal protein S4
Q2FZ31	1.39	Methylenetetrahydrofolate--tRNA-(uracil-5-)-methyltransferase TrmFO
Q2FZY4	1.28	NifU_N domain-containing protein
Q2G0P0	2.07	50S ribosomal protein L1
Q2FX80	1.86	Conserved hypothetical phage protein
Q2FX95	1.15	Ribosomal large subunit pseudouridine synthase, RluD subfamily, putative
Q2FY19	1.41	Ferric uptake regulation protein

Q2FXT1	2.1	GTPase Obg
P60430	1.52	50S ribosomal protein L2
Q2FY45	1.25	Transcription antitermination protein NusB
Q2FV11	1.7	Oxygen-dependent choline dehydrogenase
Q2FYU6	1.78	50S ribosomal protein L33 1
Q9F0R1	1.13	HTH-type transcriptional regulator SarR
Q2FZV0	1.04	Kinase
Q2FZ06	1.1	TIGR00282 family metallophosphoesterase
Q2FY46	1.28	Exodeoxyribonuclease 7 large subunit
P48940	1.49	30S ribosomal protein S7
Q2G255	1.55	N-acetyl-alpha-D-glucosaminyl L-malate synthase BshA
Q2FW29	1.37	50S ribosomal protein L36
Q2FVZ7	1.03	DNA repair/chromosome segregation ATPase
Q2FXY7	1.33	Elongation factor 4
P0A0F4	1.39	50S ribosomal protein L11
Q2G111	2.65	30S ribosomal protein S18
Q2G0S8	1.82	Putative 4-diphosphocytidyl-2-C-methyl-D-erythritol kinase
Q2FXT7	1.05	Preprotein translocase, YajC subunit
Q2G2Q3	1.78	tRNA pseudouridine synthase B
Q2FXT0	1.55	50S ribosomal protein L27
Q2FW15	1.79	30S ribosomal protein S17
Q2FW33	1.05	50S ribosomal protein L17
Q2FVN4	1.25	Exported protein
Q9EZ11	1.24	4-hydroxy-tetrahydrodipicolinate reductase
Q2FZ65	1.35	PPM-type phosphatase domain-containing protein
Q2FYT7	1.25	UPF0154 protein SAOUHSC_01338
Q2G0J7	1.33	Uracil-DNA glycosylase
Q2G1K4	1.5	Galactowaldenase
Q2G033	1.22	Glycolytic operon regulator
Q2FYV8	1.68	Thermonuclease
O34090	1.76	Porphobilinogen deaminase
Q2FW17	1.64	50S ribosomal protein L24
Q2FZ60	1.23	50S ribosomal protein L28
Q2FZ42	1.51	50S ribosomal protein L19
Q2FWB3	1.16	Mannose-6-phosphate isomerase
Q2G0F0	2.98	Alpha/beta hydrolase
Q2G2Q1	1.08	30S ribosomal protein S15
Q2FZ78	1.68	Pseudouridine synthase
Q2FXT6	1.11	Queuine tRNA-ribosyltransferase
Q2FWH0	1.39	HMA domain-containing protein
Q2FYR2	1.27	Aminoacyltransferase FemA
Q2FZ97	2.15	Transcriptional regulator MraZ
Q2G2M8	1.15	UPF0374 protein SAOUHSC_02004
Q2FY48	1.15	Geranyltranstransferase, putative

Q2FXY2	1.77	CRM domain-containing protein
Q2FYP3	1.41	Conserved virulence factor B
Q2FWL8	3.9	UPF0033 domain-containing protein
Q2G0N9	1.38	50S ribosomal protein L10
Q2G1Z7	1.53	Isoprenyl transferase
Q2FYP0	1.08	Aspartate-semialdehyde dehydrogenase
Q2FZ44	1.3	Ribosome maturation factor RimM
Q2FXL1	1.16	Probable tRNA sulfurtransferase
Q2G234	1.21	Nitric oxide synthase oxygenase
Q2FW13	1.33	50S ribosomal protein L16
Q2G2V4	4.21	Putative membrane protein insertion efficiency factor
Q2FW39	1.3	30S ribosomal protein S9
Q2FXW5	2.45	Peptidase_U32_C domain-containing protein
Q2FZL1	1.45	Aminotransferase
Q2G2Q4	1.52	Ribosome-binding factor A
Q2FWJ4	1.05	RNA polymerase sigma factor
Q2FV52	1.12	Probable transglycosylase IsaA
Q2G0I5	2.13	UPF0741 protein SAOUHSC_00580
Q2FZ29	1.22	ATP-dependent protease subunit HslV
Q2FWF5	1.9	3-hydroxyacyl-[acyl-carrier-protein] dehydratase FabZ
Q2FY16	1.68	Probable endonuclease 4
Q2FZ46	1.02	Signal recognition particle protein
Q2FYN7	1.18	2,3,4,5-tetrahydropyridine-2,6-dicarboxylate N-acetyltransferase
Q2FW64	1.08	Alkaline shock response membrane anchor protein AmaP
Q2FXQ7	1.04	ATP-dependent Clp protease ATP-binding subunit ClpX
Q2FVT1	1.3	Lysostaphin resistance protein A
Q2FXT4	1.8	Holliday junction ATP-dependent DNA helicase RuvB
Q2G265	2.44	Peptide deformylase
Q2FV53	2.35	N-acetyltransferase domain-containing protein
Q2G016	1.1	N-acetyltransferase domain-containing protein
Q9RFJ6	1.19	HTH-type transcriptional regulator rot
Q2FYG4	1.27	Heptaprenyl pyrophosphate synthase subunit A
Q2G0S0	1.3	50S ribosomal protein L25
Q2FY67	1.21	Ribonuclease Z
Q2FXT2	1.06	UPF0735 ACT domain-containing protein SAOUHSC_01752
Q2FW19	1.86	30S ribosomal protein S14 type Z
Q2G282	1.84	Peroxide-responsive repressor PerR
Q2FYG7	1.4	Nucleoside diphosphate kinase
Q2FY23	1.32	5-formyltetrahydrofolate cyclo-ligase
Q2G1U2	1.2	3-dmu-9_3-mt domain-containing protein
Q2FZZ2	1.5	Methionine import ATP-binding protein MetN 2
Q2FW07	1.63	50S ribosomal protein L4
Q2FYY8	2.01	Aluminum resistance protein
Q2FZ67	1.64	16S rRNA (cytosine(967)-C(5))-methyltransferase

Q2G091	1.11	ABC transporter, ATP-binding protein
Q2FVM7	1.4	Oxygen regulatory protein NreC
Q2FXT3	1.48	Holliday junction ATP-dependent DNA helicase RuvA
Q2FXW4	2.11	U32 family peptidase
Q93T05	1.24	DNA mismatch repair protein MutL
Q2G1J6	1.56	Capsular polysaccharide biosynthesis protein Cap8M
Q2FXX9	2.26	YqeG family HAD IIIA-type phosphatase
Q2FZV8	1.29	Fe-S_biosyn domain-containing protein
Q2FZZ3	1.34	Thioredoxin domain-containing protein
Q2G046	1.06	UvrABC system protein A
Q2FWF8	2.11	Probable transglycosylase SceD
Q2G0D8	1.94	Putative hemin import ATP-binding protein HrtA
Q2G132	1.03	Ribosomal-protein-serine acetyltransferase, putative
Q2FYQ9	1.44	SWIM-type domain-containing protein
Q2FZP4	1.58	Peptide chain release factor 3
Q2G0F4	1.29	Haloacid dehalogenase-like hydrolase, putative
Q2G202	1.36	Globin
Q2G2T9	1.09	HlyC/CorC family transporter
Q2FYN6	1.37	Uncharacterized hydrolase SAOUHSC_01399
Q2FYY5	1.29	DNA-binding protein
Q2G1T4	2.9	N-acetyltransferase domain-containing protein
Q2FXT5	1.27	S-adenosylmethionine:tRNA ribosyltransferase-isomerase
Q2FWM4	1.79	Accessory gene regulator protein A
Q2FV59	1.46	4,4'-diapophytoene synthase
Q2FV57	1.99	4,4'-diaponeurosporene oxygenase
Q9KJN4	1.68	Response regulator ArlR
Q2FZX2	1.07	Cytosolic protein
Q2G221	1.4	Phage infection protein, putative
Q2FZF1	1	50S ribosomal protein L32
Q2G0F1	1.06	DUF1129 domain-containing protein
Q2G024	2.55	Ribonuclease R
Q2FXT9	1.26	Single-stranded-DNA-specific exonuclease RecJ
Q2FZD3	2.35	Endonuclease MutS2
Q2FWX5	1.29	Linear amide C-N hydrolase
Q53726	1.12	Heptaprenylglyceryl phosphate synthase
Q2FX12	1.73	Low molecular weight protein-tyrosine-phosphatase PtpA
Q9RQQ0	2.29	Biofilm operon icaADBC HTH-type negative transcriptional regulator IcaR
Q2G1W1	1.73	Secretory antigen SsaA, putative
Q2FUY2	1.63	Clumping factor B
Q2FXR9	1.23	Folylpolyglutamate synthase/dihydrofolate synthase, putative
Q2FZ84	1.61	S4 domain-containing protein
Q2FXW3	2.17	tRNA 5-hydroxyuridine methyltransferase
Q2FXI2	4.15	tRNA (guanine-N(7)-)-methyltransferase

Q2G2A7	1.67	Spermidine/putrescine import ATP-binding protein PotA
Q2FYI7	1.21	DUF4889 domain-containing protein
Q2G0F9	1.38	DUF1934 family protein
Q2G1P4	2.29	PhoU domain-containing protein
Q2FYU4	1.3	GMP reductase
Q2FZA5	1.37	DNA-binding protein
Q2FY13	1.37	tRNA methyltransferase
Q2FWJ7	1.35	Fe-S cluster assembly ATPase SufC
Q2FY15	1.58	DEAD-box ATP-dependent RNA helicase CshB
Q2FY65	1.73	HTH araC/xylS-type domain-containing protein
Q2FZF4	2.43	tRNA(Met) cytidine acetate ligase
Q2G0V2	3.07	Methionine import ATP-binding protein MetN 1
Q2G0G5	1.63	FMN-dependent NADPH-azoreductase
Q2G097	1.84	Chorismate binding enzyme, putative
Q2G2A0	2.08	Methyltransf_25 domain-containing protein
Q2G1K1	2.6	Capsular polysaccharide synthesis enzyme O-acetyl transferase Cap5H, putative
Q2FY17	1.93	ABC transporter, putative
Q2FYT0	1.63	Glycine betaine transporter, putative
Q2G0E7	1.88	TPR_REGION domain-containing protein
Q2FZH3	1.62	Cytochrome d ubiquinol oxidase, subunit I, putative
Q2G0V1	1.78	ABC transporter, permease protein, putative
Q2FZU8	1.16	S1 motif domain-containing protein
Q2FWY3	1.08	Pectate_lyase_3 domain-containing protein
Q2FZQ4	1.41	Sodium:proton antiporter
Q2FZH2	1.46	Cytochrome d ubiquinol oxidase, subunit II, putative
Q2FZH9	1.13	Membrane spanning protein
Q2FXH5	1.96	NAD(P)/FAD-dependent oxidoreductase
Q2FW37	2.42	tRNA pseudouridine synthase A
Q2FVT7	1.61	M20_dimer domain-containing protein
Q2G0B3	1.17	ABC transporter ATP-binding protein/permease
Q2G1X3	2.81	Phage protein
Q2G2M2	1.27	Phosphatidylglycerol lysyltransferase
Q2FZX7	1.32	DUF72 domain-containing protein
Q2FW72	1.16	Staphyloferrin A synthase
Q2G037	1.33	Probable cell division protein WhiA
Q2FYK0	1.3	Probable queuosine precursor transporter
Q2G2M9	1.11	Putative multidrug export ATP-binding/permease protein SAOUHSC_02003
Q2FX93	1.16	DUF2975 domain-containing protein
Q2FVN8	1.47	Transcriptional regulator, putative
Q2G0B2	1.07	Amino acid ABC transporter ATP-binding/permease protein
Q9F1K0	1.67	DNA polymerase III subunit alpha
Q2FXY5	1.23	DNA_pol3_delta domain-containing protein

Q2FXY9	1.4	Heme chaperone HemW
Q2FZQ0	1.79	Esterase family protein
Q2FYS6	1.82	Glycerol-3-phosphate acyltransferase
Q2G0I1	1.31	LXG domain-containing protein
Q2FW34	1.46	Energy-coupling factor transporter ATP-binding protein EcfA1
Q2FWQ5	1.1	Conserved hypothetical phage protein
Q2FVP6	1.43	DUF2871 domain-containing protein
Q2G1U5	1.89	Adapter protein MecA
Q2FWV5	1.28	Chemotaxis inhibitory protein
Q2FVV1	1.31	AA_permease domain-containing protein
Q2G2C2	1.05	Cell division protein FtsW
Q2FXF2	1.53	RNA polymerase sigma factor SigS
Q2G2S9	1.03	DUF3267 domain-containing protein
Q2FWK3	4.44	2-isopropylmalate synthase
Q2G110	1.12	Abi family protein
Q2FY18	1.06	Metal ABC transporter permease
Q2G1A8	1.4	Methyltransf_25 domain-containing protein
Q2FZA8	1.2	YfcC family protein
Q2FUS8	1.04	Lactonase drp35

Supplementary table 5. The differentially expressed proteins with the down-regulated level by quantitative proteomic analysis in *S. aureus* treated with radezolid.

ID	Log2FoldChange	Description
Q2FZ07	-1.63	HEPN domain-containing protein
Q2G189	-2.03	Type VII secretion system extracellular protein A
Q2G1I8	-1.28	DUF2171 domain-containing protein
Q2FXL6	-1.13	Putative universal stress protein SAOUHSC_01819
Q2FZJ2	-2.57	Phosphoribosylformylglycinamidine synthase subunit PurS
Q2FV63	-2.89	Copper chaperone CopZ
Q2FYF9	-1.76	30S ribosomal protein S1, putative
Q2FWD3	-1.31	Fructose-bisphosphate aldolase
Q2G0K7	-1.27	3-hexulose-6-phosphate synthase
Q2FY08	-1.15	Glycine--tRNA ligase
P0A0B7	-1.25	Alkyl hydroperoxide reductase C
Q2FXI0	-2.34	D-alanine aminotransferase
Q2G041	-1.04	Thioredoxin reductase
P95689	-1.83	Serine--tRNA ligase
Q2G0Q1	-1.61	Pyridoxal 5'-phosphate synthase subunit PdxS
Q2FY40	-1.14	Proline dipeptidase, putative
Q2G0G1	-1.06	Alcohol dehydrogenase
Q2FZZ9	-1.49	Arsenate reductase family protein
Q2FYA0	-2.45	Phage-related protein
Q2FXJ6	-1.93	Serine protease HtrA-like
Q2FWX1	-2.18	Thioredoxin family protein
P0A0J3	-1.02	Superoxide dismutase [Mn] 1
Q2G1I7	-2.07	DUF4242 domain-containing protein
Q2G1F2	-1.65	FMN-dependent NADH:quinone oxidoreductase
Q2FXI5	-1.04	Glutamyl aminopeptidase
Q2FZK7	-2.27	Bifunctional autolysin
Q2FVX8	-1.12	Molybdenum cofactor biosynthesis protein B
Q2G126	-1.23	DUF1398 domain-containing protein
Q2FZM1	-1.18	N-acetyltransferase domain-containing protein
Q2G2M0	-1.27	Tautomerase
Q2FWP0	-4.81	Uncharacterized leukocidin-like protein 1
Q2FXI6	-1.52	Thioredoxin domain-containing protein
Q2FVK8	-2.9	2,3-bisphosphoglycerate-dependent phosphoglycerate mutase
Q2G2A3	-1.12	Dihydrolipoyl dehydrogenase
Q2FW49	-2.58	Acetolactate synthase, putative
Q2FX09	-1.63	Response regulator protein VraR
Q2G2A1	-1.56	CMP/dCMP-type deaminase domain-containing protein
Q2G0P5	-1.08	ATP-dependent Clp protease ATP-binding subunit ClpC
Q2G2D7	-1.54	Enoyl reductase (ER) domain-containing protein
Q2G019	-2.07	DUF5067 domain-containing protein

Q2FVK5	-5.48	Immunoglobulin-binding protein Sbi
Q2FWN3	-1.02	Co-chaperonin GroES
Q2FY98	-3.6	Conserved hypothetical phage protein
Q2G0Q0	-1.53	Pyridoxal 5'-phosphate synthase subunit PdxT
Q2G1M1	-2.94	Diacetyl reductase [(S)-acetoin forming]
Q2FXH9	-1.2	Putative dipeptidase SAOUHSC_01868
Q2FVA4	-3.17	Putative NAD(P)H nitroreductase SAOUHSC_02829
Q2G0F2	-1.88	Exported protein
Q2G2J2	-1.01	Staphylococcal secretory antigen ssaA2
Q2FX98	-1.59	HTH cro/C1-type domain-containing protein
Q2FWC1	-2.14	Pyrimidine-nucleoside phosphorylase
Q2FY01	-1.3	PhoH domain-containing protein
Q2G222	-4.17	N-acetylmuramoyl-L-alanine amidase domain-containing protein SAOUHSC_02979
Q2FW50	-1.84	Alpha-acetolactate decarboxylase
Q2FXP7	-1.72	Threonine--tRNA ligase
Q2G280	-1.5	NADH-dependent peroxiredoxin
Q2FYZ0	-1.1	Glutathione peroxidase
Q2FVV8	-1.55	Transcriptional regulator, putative
Q2FWN4	-1.47	Chaperonin GroEL
Q2G227	-1.1	Phosphopentomutase
Q2FZL5	-1.06	1,4-dihydroxy-2-naphthoyl-CoA synthase
Q2G1T3	-1.08	Organic hydroperoxide resistance protein-like
Q2FVG5	-2.1	DUF1801 domain-containing protein
Q2G1K9	-1.28	Aldehyde-alcohol dehydrogenase
Q2G0S2	-1.11	Ribose-phosphate pyrophosphokinase
Q2G0J0	-1.03	Phosphate acetyltransferase
Q2FZU0	-1.97	Glucose-6-phosphate isomerase
Q2FVY0	-1.21	Molybdopterin molybdenumtransferase
Q2G270	-1.1	bPH_3 domain-containing protein
Q2FZU7	-1.69	FMN oxidoreductase, putative
Q2FVX4	-1.45	Molybdenum ABC transporter, periplasmic molybdate-binding protein
Q2FUX3	-8.03	Immunodominant staphylococcal antigen B
Q2G2S0	-1.42	Adenylosuccinate lyase
Q2FYZ4	-1.11	Aerobic glycerol-3-phosphate dehydrogenase
Q2G2M6	-1.12	Cysteine--tRNA ligase
Q2G1U3	-1.58	Oligoendopeptidase F
Q2FWB9	-3.24	Deoxyribose-phosphate aldolase
Q2FW96	-1.05	Mannitol-1-phosphate 5-dehydrogenase
P02976	-4.25	Immunoglobulin G-binding protein A
Q2FYJ2	-1.65	Alanine dehydrogenase 1
Q2FWB8	-1.15	Purine nucleoside phosphorylase DeoD-type
Q2FVT8	-1.01	SDR family oxidoreductase

Q2G1A1	-1.08	DNA-binding protein
Q2FVA3	-3.25	D-lactate dehydrogenase
Q2G1Y5	-2.36	L-lactate dehydrogenase 2
Q2G0U7	-1.21	MutT/nudix family protein, putative
Q2G2G0	-1.87	DM13 domain-containing protein
Q2FVN6	-1.1	DUF4889 domain-containing protein
Q2G2D8	-4.06	ABC transporter, substrate-binding protein, putative
Q2G2B2	-4.41	Surface protein G
Q2FXL3	-1.22	Thiol peroxidase
Q2G0P6	-1.38	Protein-arginine kinase
Q2FYF1	-1.08	Elastin-binding protein EbpS
Q2G2P2	-1.79	nitric oxide dioxygenase
Q2FVS3	-1.34	ABC transporter domain-containing protein
Q2FZ74	-2.03	Dihydroorotase
Q2G261	-1.01	Superoxide dismutase [Mn/Fe] 2
Q2FWN9	-4.02	Uncharacterized leukocidin-like protein 2
Q2FYM9	-1.21	Acylphosphatase
O05204	-1.46	Alkyl hydroperoxide reductase subunit F
Q2FVF5	-1.33	Lipoprotein
Q2FZD8	-1.12	Phenylalanine--tRNA ligase beta subunit
Q2FVC2	-1.05	Pyrophosphohydrolase, putative
Q2FVK2	-3.21	Gamma-hemolysin component C
Q2FUR8	-2.42	Arylamine N-acetyltransferase
Q2G2K2	-1.16	Bacteriophage L54a, bacterial Ig-like domain group 2 family protein
Q2G2L7	-2.25	RelA_SpoT domain-containing protein
Q2G2P4	-3.14	DUF488 domain-containing protein
Q2FVI6	-1.05	Viral A-type inclusion protein
Q2FWB7	-1.75	Ferritin domain-containing protein
Q2G193	-1.53	Exported protein
Q2G093	-2.35	Lipoteichoic acid synthase
P0A086	-1.01	Peptide methionine sulfoxide reductase MsrA 2
Q2FY92	-2.56	Conserved hypothetical phage protein
Q2FXE2	-1.73	Aldo_ket_red domain-containing protein
Q2G087	-1.17	Histidinol-phosphate aminotransferase
Q2FY59	-2.01	Peptidase T, putative
Q2G273	-3.35	Urease accessory protein UreG
Q2G0K6	-1.12	SIS domain protein
Q2FXL9	-1.78	Uncharacterized peptidase SAOUHSC_01816
Q2FY68	-1.07	Pyrroline-5-carboxylate reductase
Q2FZZ6	-1.99	DUF1963 domain-containing protein
Q2FZZ8	-1.05	Glycine cleavage system H protein
Q2FZU3	-3.01	GP-PDE domain-containing protein
Q2FZN7	-1.51	lipoate--protein ligase

Q2FZG1	-1.54	SCP-like extracellular
Q2FYQ2	-1.18	Oligoendopeptidase F
Q2G2S2	-1.94	Conserved hypothetical phage protein
Q2FZ88	-1.52	Purine nucleoside phosphorylase
Q2G190	-3.83	Peptidase C51 domain-containing protein
Q2FZA1	-1.02	Uncharacterized N-acetyltransferase SAOUHSC_01138
Q2FZI6	-1	Bifunctional purine biosynthesis protein PurH
Q2FWZ8	-2.28	Bacterial non-heme ferritin
Q2FW57	-3.66	Aldo_ket_red domain-containing protein
Q2FVC7	-3.05	Phage protein
Q2G2U0	-1.64	N-acetylglucosamine-6-phosphate deacetylase
Q93Q23	-1.46	Monofunctional glycosyltransferase
Q2FXH2	-1.05	Leucine--tRNA ligase
Q2FWY2	-1.74	Pyrazinamidase/nicotinamidase, putative
Q2G2K3	-3.89	Conserved hypothetical phage protein
Q2FVA5	-1.39	VOC domain-containing protein
Q2FUS9	-2.12	UPF0312 protein SAOUHSC_03022
Q2G1P3	-1.52	Oleate hydratase
Q2G021	-1.46	Conserved domain protein
Q2G2K8	-3.81	Urease accessory protein UreE
Q2G135	-2.01	FMN-dependent NADPH-azoreductase
Q2FZJ3	-1.07	Phosphoribosylaminoimidazole-succinocarboxamide synthase
Q2FVG8	-1.57	Amino acid ABC transporter, ATP-binding protein, putative
Q2G1S3	-1.2	Adenylosuccinate synthetase
Q2G0U9	-1.54	N-acetylmuramoyl-L-alanine amidase sle1
Q2G067	-3.96	EMYY motif lipoprotein
Q2G000	-2.14	Thioredoxin, putative
Q2G0E2	-1.97	N-acetyltransferase domain-containing protein
Q2FZB8	-3.37	Fibrinogen-binding protein
Q2G0Y0	-1.52	NAD(P)-bd_dom domain-containing protein
Q2FZ61	-1.61	thiamine diphosphokinase
P0A011	-1	Tagatose 1,6-diphosphate aldolase
Q2G0D4	-2.36	Probable autolysin SsaALP
Q2G170	-2.9	5'-nucleotidase, lipoprotein e(P4) family
Q2FVZ2	-1.02	Glyoxalase-like_dom domain-containing protein
Q2FXE8	-1.5	Transaldolase
Q2FXG2	-2.58	6,7-dimethyl-8-ribityllumazine synthase
Q2FZJ0	-2.78	Phosphoribosylformylglycinamidine synthase subunit PurL
Q9RQP7	-4.22	Poly-beta-1,6-N-acetyl-D-glucosamine N-deacetylase
Q2G2W8	-1.42	DNA replication initiation control protein YabA
Q2FZC2	-4.36	Fibrinogen-binding protein
Q2FZ75	-2.76	Aspartate carbamoyltransferase
Q2FVC6	-4.28	Lipase
Q2G188	-2.1	Type VII secretion system accessory factor EsaA

Q2FVW5	-6.46	Urease subunit gamma
Q2G264	-3.59	Lipoprotein
Q2G2G7	-2.11	UPF0637 protein SAOUHSC_01054
Q2G0T3	-1.67	Recombination protein RecR
Q2G0Z5	-1.51	NADPH-dependent oxidoreductase
Q2FVH0	-1.05	Amino acid transporter, putative
Q2G136	-1.37	Bac_luciferase domain-containing protein
Q2FZ43	-1.3	tRNA (guanine-N(1)-)-methyltransferase
Q2FV88	-1.71	Acyl-CoA thioesterase
Q2FW56	-3.35	Transcriptional regulator, merR family, putative
Q2G155	-3.95	Lipase 2
Q2FZ73	-1.59	Carbamoyl-phosphate synthase small chain
Q2FZS2	-2.5	Truncated MHC class II analog protein
Q2G2E9	-2.53	TIGR04141 family sporadically distributed protein
Q2G2D6	-1.58	Alpha/beta hydrolase
Q2FZU1	-1.16	Argininosuccinate synthase
Q2G1W2	-1.36	Phosphoenolpyruvate carboxykinase (ATP)
Q2FZV6	-1.66	Probable cytosol aminopeptidase
Q2FYU2	-2.96	CAP domain-containing protein
Q2FXG0	-1.97	Riboflavin synthase, alpha subunit
Q2FWS5	-1.62	Conserved hypothetical phage protein
Q2FUX8	-1.6	Ornithine carbamoyltransferase
Q2FWQ1	-1.57	Anti repressor
Q2G038	-1.51	Gluconeogenesis factor
Q2G1D7	-1.73	Pyruvate formate-lyase-activating enzyme
Q2FUY9	-1.52	DNA-binding response regulator, putative
Q2FVQ5	-1.26	Probable malate:quinone oxidoreductase
Q2FV81	-2.17	LysM domain protein
Q2FWV6	-5	Staphylococcal complement inhibitor
Q2G0X5	-5.78	Restriction modification system specificity subunit, putative
Q2FYD5	-2.09	Conserved hypothetical phage protein
Q2FZ12	-1.42	HTH cro/C1-type domain-containing protein
Q2FX07	-1.18	DUF2154 domain-containing protein
Q2FZ72	-1.11	Carbamoyl-phosphate synthase large chain
Q2G0W8	-2.97	Lipase_3 domain-containing protein
Q2G1R8	-1.71	Persulfide-sensing transcriptional repressor CstR
Q2FV34	-1.03	Type II secretion protein
Q2FWU9	-1.99	Conserved hypothetical phage protein
Q2FV19	-1.02	Alpha-acetolactate decarboxylase
Q2FUZ8	-1.93	Sulfite reductase (NADPH) flavoprotein alpha-component, putative
Q2FXK3	-2.32	SACOL1771 family peroxiredoxin
Q2G2U3	-1.55	YycH domain-containing protein
Q2G0X1	-1.06	Uncharacterized lipoprotein SAOUHSC_00402

Q2FV87	-1.88	PTS system glucoside-specific EIICBA component
Q2G1D3	-5.03	Coagulase
Q2FV54	-1.24	O-acetyltransferase OatA
Q2G2W1	-1.86	Drug resistance transporter, EmrB/QacA subfamily, putative
Q2G1M2	-2.04	MFS transporter
Q2FVG3	-1.4	Carboxylic ester hydrolase
Q2G2K1	-1.13	Major tail protein
Q2G2Y3	-1.75	DUF4352 domain-containing protein
Q2FVD5	-1.51	Uncharacterized oxidoreductase SAOUHSC_02778
Q2G1P9	-1.02	Uncharacterized lipoprotein SAOUHSC_00054
Q2G122	-1.06	5-methyltetrahydropteroyltriglutamate--homocysteine methyltransferase
Q2FVD2	-2.89	ATP phosphoribosyltransferase
Q2FY86	-1.15	Conserved hypothetical phage protein
Q2FX08	-1.5	Sensor protein VraS
Q2FYA8	-1.23	PVL orf 52-like protein
Q2FVH1	-1.66	Amino acid ABC transporter, permease protein, putative
Q2FZR3	-1.13	Oligopeptide ABC transporter, substrate-binding protein, putative
Q2G2G3	-2.46	Divalent metal cation transporter MntH
Q2FVW2	-1.68	N-acetylmuramoyl-L-alanine amidase, putative
Q2FYB1	-1.26	PVL orf 52-like protein
Q2G1H7	-2.51	DUF1440 domain-containing protein
Q2FWA6	-1.75	Truncated resolvase
Q2FZ56	-1.21	Transcription factor FapR
Q2G2R8	-1.66	Staphopain A
Q2G2E4	-1	NupC/NupG family nucleoside CNT transporter
Q2G2P5	-1.83	Nickel-binding protein NikA
Q2G2F4	-2.98	ATP synthase subunit a
Q2FWN2	-1.07	Abortive infection protein
Q2FYE3	-1.22	Lipoprotein
Q2G0L4	-2.34	Serine-aspartate repeat-containing protein D
Q2G179	-1.98	Type VII secretion system protein EssD
Q2FZI8	-2.18	Phosphoribosylformylglycinamidine cyclo-ligase
Q2FV24	-1.34	PepX_C domain-containing protein
Q2G2M4	-3.76	Mini-ribonuclease 3
Q2FZM5	-2.47	DoxX family protein
Q2FXG1	-1.4	Riboflavin biosynthesis protein RibBA
Q2G1J0	-2.35	Putative aldehyde dehydrogenase AldA
Q2G0J3	-1.07	ThrE_2 domain-containing protein
Q2FVG9	-1.52	Amino acid ABC transporter, permease protein, putative
Q2FVN1	-1.14	Probable nitrate transporter NarT
Q2FV70	-1.66	SSD domain-containing protein
P14738	-3.54	Fibronectin-binding protein A

Q2G0L5	-1.94	Serine-aspartate repeat-containing protein C
Q2G134	-1.18	YeiH family protein
Q2FUW1	-1.73	Serine-rich adhesin for platelets
Q2FVX5	-3.99	Molybdenum transport system permease
Q2FW83	-1.1	MFS domain-containing protein
Q2G1M9	-1.44	L-2,3-diaminopropanoate--citrate ligase

Supplementary table 6. The relationship between the MIC values of radezolid, contezolid and linezolid against a minority of *S. aureus* isolates.

strains	Radezolid (mg/L)	Contezolid (mg/L)	Linezolid (mg/L)
YUS1A145	0.5	2	2
YUSA110	0.25	2	2
YUSA101	0.125	0.5	0.5
YUSA138	0.125	0.5	1
YUSA61	0.25	1	1
YUSA16	0.5	2	1
YUSA21	0.25	2	2
YUSA31	0.0625	1	0.5
YUSA33	0.25	2	1
YUSA84	0.5	2	1
YUSA95	0.25	2	2
CHS101	0.25	4	2
CHS707	0.5	4	2
CHS709	0.25	2	2
CHS712	0.5	2	1
CHS736	0.125	1	0.5
